# Brain linkage for good governance: the need for Guatemalan diaspora expertise

**DOI:** 10.3389/frma.2026.1856460

**Published:** 2026-07-31

**Authors:** Kleinsy Bonilla, Diego Alejandro Quan Reyes, Natalia Ortíz-Barrientos, Antonio Romero, Annelisse Fernández Moreno

**Affiliations:** 1 Observatorio Económico Sostenible, Universidad del Valle de Guatemala, Guatemala City, Guatemala; 2 Organization for Women in the Developing World, Guatemala National Chapter, Guatemala City, Guatemala; 3 Mechanical Engineering, Eindhoven University of Technology, Eindhoven, Netherlands; 4 Independent Researcher, Guatemala City, Guatemala; 5 Department of Solid Waste Management, Ministry of Environment and Natural Resources MARN, Guatemala City, Guatemala

**Keywords:** brain linkage, diaspora engagement, good governance, Guatemala, international mobility, migration, scientific diaspora, corruption

## Abstract

The Guatemalan scientific and professional diaspora (GSPD) is composed of highly educated Guatemalans who have established their residence abroad, are engaged in professional activities in their host country, and have active links with their country of origin. As a group, they represent an undervalued and publicly unrecognized asset for strengthening public governance and institutional performance in their home country. The existing public administration in Guatemala has been historically undermined by poor governance, corruption, and influential vested interest groups that have systematically hindered the development of a robust public sector. The lack of qualified human resources fuels poor government performance. On the one hand, government employment systems are dysfunctional and underdeveloped. On the other hand, there is insufficient and inadequate local expertise, which is difficult to hire in the public administration due to low salaries, unstable labor conditions, and hostile working environments. Furthermore, the underdevelopment of the local higher education system and chronically low investment in education have further resulted in an inadequate supply of highly educated workers. Yet, a Guatemalan scientific and professional diaspora numbering in the thousands is already making available its expertise to its home country, although dispersed and uncoordinated. Recognizing, engaging, and coordinating these highly skilled Guatemalans in the diaspora can significantly improve governance and institutional performance at both national and local levels. In this Perspective, the authors reflect on this intersection, drawing from their first-hand experience as members of the GSPD and as practitioners within public institutions.

## 1 Introduction

**Figure 1:**
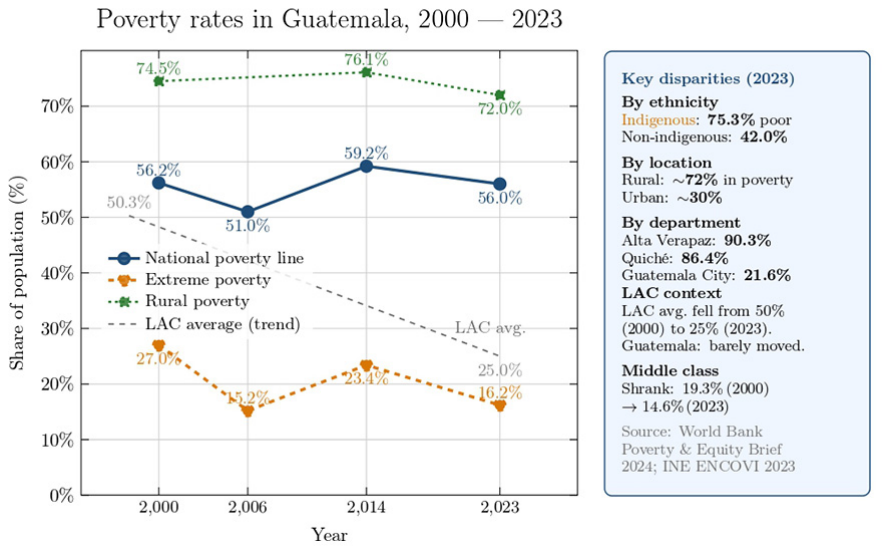
Poverty, inequality and ethnic divide in Guatemala.

Guatemala is the largest economy in Central America, yet its economic model remains anchored in low-value adding, labor-intensive primary and agro-industrial exports. Bananas, coffee, raw sugar, and palm oil rank among the country's leading export products and account for a substantial share of export revenues (Cabrera et al., 2015). The underlying political-economic configuration produces extreme concentration of wealth, resources, and political power. The poverty rates in Guatemala, contrasted with the Latin-American average shown in Figure 1, demonstrate the failure of the current economic model in improving the life-quality of Guatemalans, and its success in maintaining rural and Indigenous communities excluded from economic development. This is also reflected in Guatemala being among the most unequal countries in Latin America, with its wealthiest decile holding close to 50% of national income (Cabrera et al., 2015). This concentration limits economic diversification, constrains competition and productivity, and reduces incentives for scientific and technological advancement.

Figure 1 Poverty rates in Guatemala, 2000–2023. National poverty is measured at the official Guatemalan poverty line (close to the World Bank upper-middle-income poverty threshold of US$6.85/day, 2017 purchasing power parity). Extreme poverty corresponds to the minimum caloric intake cost. Rural poverty is shown for the years in which rural-specific estimates are available. Some key disparities are shown in the right box.

**Figure 2:**
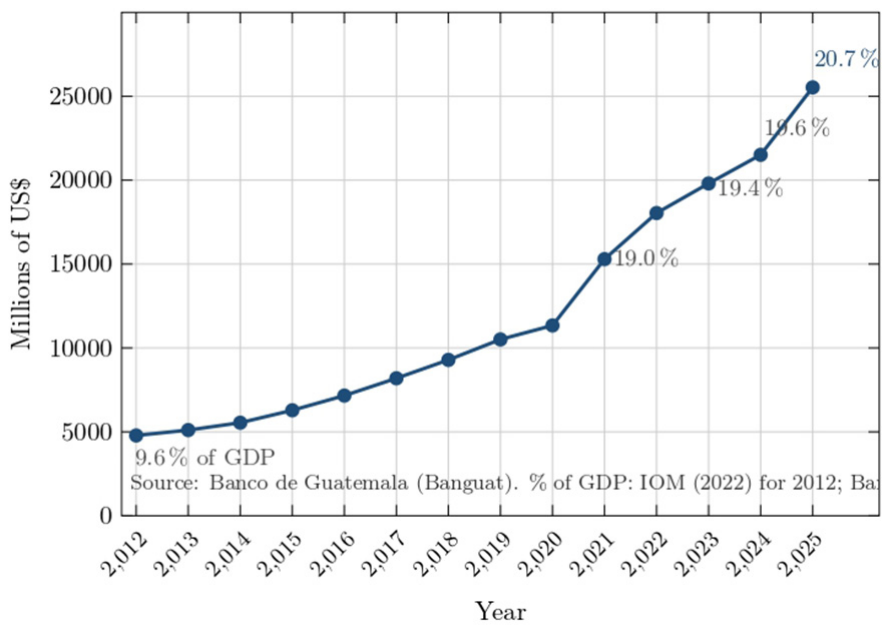
Incoming remittances to Guatemala 2012–2025.

Guatemala's economy is further shaped by remittances, as can be seen in the historical evolution shown in Figure 2. By 2025, remittances had reached a historic share of national income, amounting to approximately 20% of GDP (Banco de Guatemala, 2026). These flows are primarily directed toward consumption and the coverage of basic needs. In essence, the difficult living conditions within the country drive emigration, which generates billions in remittances that ultimately continue sustaining the unequal economic and political structures that produced those conditions in the first place.

Emigration toward the United States of America (USA) has been persistent throughout the second half of the twentieth century, and the pace has accelerated over the last two decades. While Guatemala has a population of over 18 million in the territory, an estimated 3.5 million have migrated and reside overseas. According to the Guatemala Migration authority (Autoridad Migratoria Nacional, 2023) the Guatemalan population residing abroad is concentrated in the United States (95.4%), followed by Mexico (2.45%) and Belize (1.01%), with the remaining 1.14% distributed across other countries.

These structural conditions, combined with a fragile and still-emerging democratic system, have produced a fragmented and underdeveloped national system of science, technology, and innovation. This is reflected in Guatemala's expenditure on research and development, which has remained at approximately 0.06% of GDP (World Bank, 2025), a figure representing roughly one-fiftieth of the world average, and a structural impediment to national innovation. Public spending on education has also been stagnant, hovering around 3.2% of GDP for more than a decade (UISUNESCO Institute for Statistics, 2024; International Monetary Fund, 2024). This is the lowest level of spending in the Central American region and far below the global benchmark of 4%. Secondary education coverage reflects the same pattern: only about 44% of adolescents are enrolled (compared to a world average of over 80%), with particularly acute exclusion among rural, poor, and Indigenous populations. This limits the formation of an adequately skilled labor force and reproduces territorial inequalities. At the tertiary level, according to the most recent available UNESCO data, only approximately 7% of the population aged 25 and over has attained a bachelor's degree or equivalent, one of the lowest rates in the region (UISUNESCO Institute for Statistics, 2024; International Monetary Fund, 2024). In all, to understand the education level of Guatemala's labor force, it helps to visualize a steep pyramid: a broad base with some primary school experience, a rapidly shrinking middle with secondary education, and a very small tip of highly educated university graduates. In this context, it becomes a paradox how the existence of rigid legal and administrative frameworks, particularly in government, regarding the incorporation of external knowledge resources, technical capabilities, and infrastructure, limits the possibility of integrating diaspora expertise that does not conform to established administrative procedures. The absence of a coherent policy framework further constrains the scale and sustainability of diaspora engagement, reflecting a structural disconnect between migration governance and science and technology policy. Another structural factor that contributes to this dire context is the aforementioned poor governance in Guatemala. The fiscal cost of corruption compounds these structural deficits in ways that are difficult to fully quantify but impossible to ignore. *Analysts at the Instituto Centroamericano de Estudios Fiscales* (ICEFI) and *Acción Ciudadana* (Transparency International Guatemala) agree that a precise, unified figure is impossible to establish, given how much corruption remains hidden (Instituto Centroamericano de Estudios Fiscales (ICEFI) Oxfam Guatemala, 2015). That said, the available estimates are striking. Acción Ciudadana's study on fiscal impunity and corruption calculates losses at approximately 24% of the National Budget annually, while a parliamentary analysis from the same period estimated the loss to be around 20% of public investment.

Despite the structural constraints, a highly skilled Guatemalan scientific and professional diaspora (GSPD) has emerged, mostly trained and educated in prestigious universities around the world supported by international cooperation, scholarships and other forms of aid (Bonilla et al., 2025; Bonilla and Kwak, 2015). This GSPD is contributing to its home country -in spite of multiple barriers-with information and knowledge management, transnational network building, institutional capacity building and multi-actor coordination, as well as strategic advice and policy influence. Admittedly, these contributions are still dispersed and through largely uncoordinated initiatives (Bonilla et al., 2022b; Echeverría King et al., 2025; Moshtari and Ghorbani, 2025). In this context, the willingness of the scientific and professional diaspora to contribute knowledge, experience, and expertise to Guatemala lacks a commensurate institutional counterpart. The principal obstacle is the absence of flexible mechanisms that would allow for the recognition, reception, and operationalization of such contributions. This represents a concrete loss of public value, as it restricts the diaspora's role as a strategic actor in strengthening governance and evidence-based decision-making. The following sections examine what the literature tells us about contributions of science and professional diasporas to their countries of origin, analyze the institutional setting shaping diaspora engagement in Guatemala, and make the case for a closer look at the role that highly educated Guatemalans residing abroad could play toward good governance at home.

## 2 Contributions from science and professional diasporas

Among the possible solutions to offset the effects of brain drain on countries of origin, one is to harness the skilled diaspora's transnational social capital, that is, the networks of relationships among individuals who live and work within a given society and across different cultures and geographies (Shin and Caywood, 2025). This approach, termed brain linkage, recognizes that emigrants can support their home country's development by drawing on this transnational social capital without permanently returning. On the governmental side, a growing number of agencies in different countries now focus on immigrants in response to high-skilled mobility. Some interventions adopt a brain linkage approach, seeking to leverage diaspora resources and strengthen transnational communities. In a systematic review of the science, technology, and innovation (STI) impacts of scientific diasporas, Ezekiel (2023) classified diaspora engagement with countries of origin into the following categories:

- Capacity building and education: collaboration between diaspora scientists and home-country educational institutions through mentorship programs and initiatives to strengthen research capabilities in STEM and innovation.
- Building science-based international partnerships: initiatives aimed at establishing international cooperation for STI development.
- Technology transfer, STI infrastructure, and institutional capacity development: contributions intended to enhance technological infrastructure and strengthen STI capacity in home countries.
- Science advice: the provision of expertise to inform national STI-related policies.
- Donation of scientific and related equipment: contributions to home institutions through scholarly articles, equipment, and infrastructure.

Within each of these categories, interventions vary according to the specific circumstances of diasporas and home countries. As Congreso de la República de Guatemala (1991) note, effective engagement models combine multiple modalities, including mentoring, technical advice, short visits, and remote institutional collaboration. Examples of interventions include consultancies, teaching, research, scholarships, binational projects with reciprocal visits, short workshops led by diaspora members followed by online mentoring, virtual mentoring for startups, in-person hackathons, and platforms that profile members and offer optional roles such as mentor, evaluator, investor, or visiting scholar, often under flexible contracts.

Given the wide range of possibilities for mobilizing a diaspora's transnational social capital, a central challenge is determining which interventions are feasible within a given national context. Engagement strategies range from small-scale projects to highly structured programs and require a long-term perspective to yield results (Aikins and White, 2011). Moreover, diaspora-linking initiatives do not necessarily require direct government involvement. Scientific diaspora organizations may also emerge organically through bottom-up processes initiated by diaspora members themselves. Such approaches appear to demonstrate greater sustainability than top-down, government-led initiatives (Ezekiel, 2023).

Aikins and White (2011) identify several key lessons for developing effective and sustainable diaspora strategies:

- Leverage global interconnectedness by creating electronic portals that facilitate connections among individuals, groups, and their countries of origin.
- Identify and connect exceptional individuals and organizations in both the diaspora and the home country; even a small number of highly capable actors can have a significant impact.
- Cultivate personal relationships to strengthen linkages; face-to-face interactions are particularly important for moving from transactional exchanges to durable partnerships.
- Engage leading diaspora members in time-bound, clearly defined group projects to sustain motivation and commitment.
- Governments should prioritize facilitation rather than direct implementation. Their role should be to endorse and support diaspora initiatives, recognize their potential contributions, and invite participation in relevant forums and policymaking processes.

## 3 Guatemala's institutional setting for diaspora engagement

As a country of origin, transit, and destination within the Central-North American migration corridor, Guatemala has undertaken binding international commitments in migration governance and human rights. The adoption of the global compact for safe, orderly and regular migration commits the country to promoting diaspora engagement as a driver of sustainable development, including through knowledge transfer and civic participation (United Nations General Assembly, 2018). At the national level, the Migration Code (Congreso de la República de Guatemala, 2016) establishes the Guatemalan Migration System, which integrates local and national institutions within a coordinated framework for migration governance. This system coexists with the National System of Science and Technology, implemented by the National Secretariat of Science and Technology (SENACYT) (Congreso de la República de Guatemala, 1991). Within this framework, the Migration Policy's component on Migration and Development recognizes the diaspora as a strategic development actor and promotes linkages between migration and national development. This component is led by SENACYT in coordination with the Ministry of Foreign Affairs (MINEX) and the International Organization for Migration (IOM) (OIM-MINEX International Organization for Migration (IOM) and Ministry of Foreign Affairs of Guatemala (MINEX), 2022; Autoridad Migratoria Nacional, 2023). However, the limitations to strategically engaging with the scientific diaspora are not solely budgetary or rooted in inter-institutional coordination failures. There are also less visible difficulties associated with an administrative culture that place excessive emphasis on control and oversight mechanisms (Bonilla et al., 2022a). While such mechanisms are intended to prevent and address corruption, in practice, they frequently result in institutional inaction. Public servants often prioritize minimizing administrative and personal risks over innovation and the activation of new forms of collaboration. In this environment, procedures, audits, and formal requirements have shifted institutional attention away from the substantive value that the diaspora can add and toward strict compliance with regulations that are frequently impractical and outdated. The result is a set of lengthy, fragmented processes that hinder the rapid integration of specialized knowledge, technical advice, or temporary collaborative arrangements. Institutional efforts targeting the diaspora remain primarily focused on social, cultural, and entrepreneurial engagement, with limited emphasis on scientific collaboration.

Although national policy mandates actions to link scientists abroad, implementation is constrained by low investment and fragmented coordination (Bonilla et al., 2022b). Initiatives such as EducaCTI^1^ and Converciencia^2^ demonstrate tangible progress in knowledge transfer (SEGEPLAN Secretariat of Planning and Programming of the Presidency, 2026). SENACYT also drives the Guatemala International Science, Technology, and Innovation network RedCTI^3^ to map out and re-engage global talent. These platforms allow Guatemalan scientists abroad to co-develop research, advise on public policy, and provide digital innovation strategies. Integrating the efforts of Guatemalan scientific diaspora members with their local peers is transitioning from informal networking into structured, institutional forms. Because setting up physical labs in Guatemala can be restricted by budget limitations, collaboration relies on highly creative digital networks, hybrid research, and science diplomacy; yet structural limitations persist, including a reduced science budget and weak program sustainability (No-Ficción, 2025). Moreover, there appears to be an incompatibility for the local institutional absorption of the GSPD. A myriad of specialized, technical, and complex bureaucratic steps result in an unbearable administrative burden. This represents a systemic hindrance: even when Guatemalan experts residing overseas offer their capacity, time, and expertise, government institution requirements become an obstacle. For example, the homologation (recognition) of foreign-obtained academic degrees is entrusted to the only public university in Guatemala (Bonilla and Kwak, 2014), or the compulsory membership to one of the fourteen professional associations (Tobar, 2011). These steps are in the way to recognize their knowledge as valid for advice to the decision-maker. In addition, there are control systems in place in public institutions in the form of extensive manuals, creating administrative rigidity. This discourages high-level, specialized scientific participation in the public sector. While process auditors in public institutions constantly review evidence-based reports, they confuse functional attributions—the legal authorization to do something—with specialized technical capabilities—the scientific capacity to do something. This creates a critical risk when making decisions, because any scientific contribution that is not conclusive must be disguised with controls that prioritize the traceability of written documentation (Fernandez-Moreno, 2025). This rigidity manifests itself in the demand for reports that value form over substance, turning the expert into a “form filler” rather than a generator of solutions.

## 4 Brain linkage for good governance: the need for Guatemalan diaspora expertise

Against this backdrop, the Guatemalan Scientific and Professional Diaspora (GSPD) emerges as a strategic yet insufficiently integrated actor for strengthening governance and development in Guatemala. For the purposes of this article, GSPD refers to individuals who satisfy four criteria: they are nationals of Guatemala, have emigrated and established their residence abroad for at least 1 year; are highly educated (postgraduate education); engaged in academic, research, or professional activities in their host country, and maintain links with Guatemala (or have the potential and willingness to establish them) so that their expertise can produce effects there. Several countries in Latin America have developed policy frameworks that recognize and facilitate contributions from their scientific and professional diasporas, including Argentina (Pombo, 2025), Brazil (Ferreira and Gimenez, 2025), Mexico (Hernández Mondragón and Cuapio, 2025), and Colombia (Piñeros-Ayala, 2025). In contrast, Guatemala's engagement with the GSPD remains fragmented and weakly institutionalized (Bonilla et al., 2022a,b). Nevertheless, the GSPD has contributed through dispersed but significant initiatives in innovation, knowledge transfer, and policy support, demonstrating its capacity to address critical gaps in technical expertise and evidence-based decision-making.

Brain linkage provides an opportunity to draw from the diaspora's expertise and transnational social capital to expand the Guatemalan government's talent pool and advance policies requiring specialized knowledge. Through diverse modalities of transnational collaboration, brain linkage offers a valuable addition to the government's talent portfolio (Shin and Gordon, 2024), which, as noted earlier, is far from meeting expectations due to several limiting factors affecting public administration. Moreover, the diaspora's independence from the pressures and restrictions of the country of origin facilitates their involvement in transformative interventions that challenge the status quo, as illustrated by the two examples described below (Newland and Patrick, 2004). While the contributions of the diaspora might only provide a fraction of the expertise needed to improve the country's governance and poverty indicators, they can still be significant. In addition to their specialized knowledge and skills gained abroad, the GSPD's experience living in established democracies exposes them to the gaps that need to be filled in their country of origin to improve governance indicators and overall development. This combination of knowledge and experience positions the diaspora in a privileged place to advance transformative action.

As for the proposed connection between diaspora expertise to foster good governance, one of the greatest obstacles for the latter in developing or fragile states is citizen suppression, which is how local experts often face censorship, legal retaliation, or physical threats when challenging corrupt systems. In this sense, because the diaspora operates outside the physical and legal jurisdiction of the home government, they have the unique freedom to speak out and expose institutional misconduct. High-skilled diaspora networks can also bridge with host-country governments and international organizations to channel foreign aid, trade deals, or development loans on governance benchmarks, human rights records, and transparency. Good governance requires competent bureaucratic management. When diaspora professionals engage with home institutions—either permanently or through temporary expert networks they inject technical expertise that bypasses local patronage networks. In addition, because their professional identities and financial security are built abroad based on merit, these individuals are less reliant on local political godfathers or corrupt networks to advance, allowing them to make more objective, data-driven decisions.

### 4.1 The case of the “magic formula” in the sanitation of the Amatitlán Lake

The mobilization of Guatemalan and international scientists during the 2012–2016 administration around the government's proposed sanitation project for Lake Amatitlán illustrates the critical role that the scientific community can play as a technical counterweight to public decision-making processes that lack scientific rigor (Ministerio Público de Guatemala, 2016; CICIG Comisión Internacional contra la Impunidad en Guatemala, 2018). Scientific actors organized their response across three interconnected fronts. First, experts from both within the country and abroad publicly questioned a “*magic formula*” because the substance proposed to sanitize the heavily polluted Amatitlán Lake had no generic name, no sanitary registration, and no supporting scientific studies. They demanded a moratorium on its application, given uncertainty about its ecosystem effects. Second, following sustained criticism, forensic chemical analysis established that the substance consisted of a saline solution with minimal organic components, including trace amounts of hydrogen peroxide. Scientists concluded it simply lacked the capacity to treat the pollution of a hyper-eutrophic body of water such as Lake Amatitlán. Third, the scientific community and environmental organizations denounced the Ministry of Environment and Natural Resources (MARN) for disregarding the mandatory environmental impact studies required before any chemical substance could be introduced into the lake (Ministerio Público de Guatemala, 2016). These public interventions proved consequential, contributing directly to the decisions of the Public Prosecutor's Office and the International Commission against Impunity in Guatemala (CICIG) to initiate criminal investigations for fraud. In 2018, the High-Risk Court “C” sentenced former Vice-President Roxana Baldetti to 15 years and 6 months in prison (along with nine co-defendants) for leading the criminal structure that defrauded the Guatemalan state of three million USD (22.83 million Quetzals) through the fraudulent sanitation contract (CICIG Comisión Internacional contra la Impunidad en Guatemala, 2018).

### 4.2 Handling of the COVID-19 sanitary crisis

During the COVID-19 pandemic, Guatemala's scientific community played a fundamental role in the technical and health response, frequently filling critical gaps left by the public system. During 2020–2021, the academic sector prioritized research and development expenditure toward health. In 2020, 88% of academic research funds were directed toward environmental protection and health, a proportion adjusted in 2021 to focus 61.6% specifically on human health in response to the ongoing emergency. Two members of the GSPD made particularly notable contributions to this response. Dr. Gabriela Asturias Ruiz, a Guatemalan scientist trained at UVG and long-time resident of California, USA, led a team of four Guatemalan nationals (resident both abroad and in-country) to co-develop ALMA, the *Asistente de Log*í*stica Médica Automatizada*, a digital virtual assistance platform designed to counter COVID-19 misinformation and support navigation of healthcare services (Alvarado et al., 2022). The ALMA initiative illustrates how the scientific diaspora can open avenues for professionals to contribute meaningfully to Guatemala regardless of their country of residence. A second example is Dr. Edwin Asturias, a Guatemalan-born physician trained at the Universidad de San Carlos and with postgraduate specialization in pediatrics at the University of Colorado School of Medicine and in Pediatric Infectious Diseases at the Johns Hopkins School of Medicine. In May 2020, by presidential decree, he was appointed Executive Director of the Presidential Commission for Attention to the COVID-19 Emergency (COPRECOVID), where he led the national containment strategy, expanded the country's diagnostic capacity, and coordinated a multi-sector response until December 2020 (NoFiccion, 2020). These examples of diaspora contribution are in stark contrast with multiple cases of corruption from government officials still pending investigation (US Department of the Treasury, 2023).

## 5 Conclusions

Guatemala faces acute challenges in building the conditions necessary for a sufficient supply of highly educated human resources. Decades of chronic underinvestment in public education and in science, technology, and innovation have produced a structural deficit in national human capital. One direct consequence is that Guatemala's persistently elevated levels of emigration continue to drain the country of its most educated citizens, further depleting the available domestic pool. Yet this outflow need not be irreversible in its effects. There is substantial international experience documenting a diversity of interventions (involving public, private, and civil society actors) that successfully connect skilled diasporas with their countries of origin and make diasporic resources available for national development. The examples examined in this article show that GSPD contributions can be consequential. In health, they supported the design of a national pandemic containment strategy and the development of a digital health communication platform. In environmental governance, they constituted a critical technical counterweight against scientifically unfounded public policy. These cases point to a diaspora that is willing and able to contribute, even in the absence of adequate institutional mechanisms to receive, recognize, and sustain those contributions. Two arguments converge to make a more systematic engagement agenda urgent. First, Guatemala's locally formed pool of highly specialized expertise remains insufficient for the demands of modern public administration and evidence-based policymaking. Second, the GSPD has demonstrated both the willingness and the capacity to contribute to national governance in meaningful ways, but it does so without the enabling conditions needed to scale and sustain those contributions.

Part of the engagement agenda could be to advance a more integrated scientific diplomacy approach, one that embeds science and technology within foreign policy to support evidence-based decision-making and international cooperation, while fostering collaboration among government, academia, and the scientific diaspora to channel knowledge toward national development (Bonilla et al., 2022a,b; Echeverría King et al., 2025). In addition, it would be crucial to increase the capacity of government institutions to design and implement more flexible processes for contracting specialized advisory services, so that diaspora expertise can be mobilized rapidly, rather than being blocked by excessively rigid administrative frameworks. Moving from episodic to systematic engagement requires identifying the key factors behind successful interventions in the Guatemalan context, as well as the institutional arrangements, incentives, and coordination mechanisms needed to bring brain linkage to its full potential. This in turn means clarifying what roles local partners, diaspora members, and government actors are best suited to play, and designing flexible, legally viable pathways for sustained collaboration that do not replicate the administrative rigidities that currently block possibilities for further engagement. It is critical that the Guatemalan public sector modernize its processes, validate scientific information, and streamline bureaucracy.

## Data Availability

The raw data supporting the conclusions of this article will be made available by the authors, without undue reservation.

## Footnotes

1 EducaCTi-Program for science education and training funded by the National Fund of Science and Technology (https://www.youtube.com/watch?v=RwiT5yl6tdM).

2 Converciencia-Annual activity implemented by the National Secretariat of Science and Technology SENACYT and the Guatemala Science, Technology and Innovation Network RedCTi since 2005 (https://www.frontiersin.org/journals/research-metrics-and-analytics/articles/10.3389/frma.2022.898496/full).

3 The International Network of Science, Technology and Innovation of Guatemala established in 2005 (https://redcti.senacyt.gob.gt/).

## References

[B1] Aikins K. White N. (2011). Global Diaspora Strategies Toolkit. Dublin: Diaspora Matters. Available online at: https://www.idiaspora.org/sites/g/files/tmzbdl2361/files/resources/document/diaspora-toolkit-book.pdf (Accessed March 23, 2026).

[B2] Alvarado J. R Lainfiesta X. Paniagua-Avila A. Asturias G. (2022). Developing a digital technology system to address COVID-19 health needs in Guatemala: a scientific diaspora case study. Front. Res. Metr. Anal. 7:899611. doi: 10.3389/frma.2022.899611 35937848 PMC9354487

[B3] Autoridad Migratoria Nacional (2023). Política Migratoria de Guatemala. Guatemala City: Autoridad Migratoria Nacional. Available online at: https://igm.gob.gt/wp-content/uploads/2023/12/FINAL-Politica-Migratoria-interiores-1_compressed.pdf (Accessed March 23, 2026).

[B4] Banco de Guatemala (2026). Departamento de Estadísticas Macroeconómicas, Sección de Estadísticas de Balanza de Pagos. Guatemala City: Banco de Guatemala. Available online at: https://banguat.gob.gt/page/anos-2002-2026 (Accessed April 14, 2026).

[B5] Bonilla K. Arrechea S. Velásquez Pérez L. G. (2022a). Connecting scientists residing Abroad: a review of converciencia as a practice to engage the Guatemalan scientific diaspora from 2005–2020. Front. Res. Metr. Anal. 7:898496. doi: 10.3389/frma.2022.898496 35832744 PMC9271891

[B6] Bonilla K. Barrientos N. O. Contreras M. A. S. (2025). Knowledge management and the power of communication: INDESGUA as a social technology enabling equitable access to scholarships in Guatemala. Front. Commun. 10:1510024. doi: 10.3389/fcomm.2025.1510024

[B7] Bonilla K. Kwak J. S. (2014). Challenges of highly educated human resources in Guatemala. Asian J. Lat. Am. Stud. 27, 17–43. doi: 10.22945/ajlas.2014.27.3.17

[B8] Bonilla K. Kwak J. S. (2015). Effectiveness of donor support for capacity development in Guatemala: a study of scholarship provision for overseas postgraduate education. Iberoamericana 1, 293–344

[B9] Bonilla K. Romero-Oliva C. S. Arrechea S. Ortiz Osejo N. Y. Mazariegos S. Alonzo M. . (2022b). Engaging the Guatemala scientific diaspora: the power of networking and shared learning. Front. Res. Metr. Anal. 7:897670. doi: 10.3389/frma.2022.897670 35755144 PMC9215311

[B10] Cabrera M. Lustig N. Morán H. E. (2015). Fiscal policy, inequality, and the ethnic divide in Guatemala. World Dev. 76, 263–79. doi: 10.1016/j.worlddev.2015.07.008

[B11] CICIG Comisión Internacional contra la Impunidad en Guatemala (2018). Former Vice President Roxana Baldetti Sentenced for Lake Amatitlán Case. Guatemala City: CICIG. Available online at: https://www.cicig.org/case-information/c_amatitlan-lake/former-vice-president-roxana-baldetti-sentenced-for-lake-amatitlan-case/ (Accessed March 23, 2026).

[B12] Congreso de la República de Guatemala (1991). Decreto 63-91: ley Nacional de Ciencia y Tecnologí*a*. Guatemala City: Diario de Centroamérica.

[B13] Congreso de la República de Guatemala (2016). Decreto 44-2016: código de Migración. Guatemala City: Diario de Centroamérica.

[B14] Echeverría King L. Pantović B. Bonilla K. Moreno García D. (2025). Bridging Knowledge Frontiers: a Comparative Analysis of International Strategies and Methodologies to Engage Scientific Diasporas and Foster Transnational Collaboration. Barranquilla: Policy document, Universidad Simón Bolívar and International Development Research Centre (IDRC). doi: 10.17081/r.book.2025.10.17024

[B15] Ezekiel I. P. (2023). A systematic review of science, technology, and innovation impact of scientific diasporas and the role of Foreign missions. ASRIC J. Soc. Sci. Hum. 4, 30–43.

[B16] Fernandez-Moreno A. (2025). Email to Diego Quan. Personal Communication.

[B17] Ferreira G. G. Gimenez A. M. (2025). Policies on Diaspora in Brazil. Barranquilla: Technical report, Universidad Simón Bolívar and International Development Research Centre (IDRC).

[B18] Foro Social de la Deuda Externa y Desarrollo de Honduras (FOSDEH). (2024). 33 Años de Corrupción en Guatemala. Available online at: https://biblioteca.fosdeh.hn/wp-content/uploads/2025/10/2024-PP-INV-055.pdf (Accessed March 23, 2026).

[B19] Hernández Mondragón A. C. Cuapio A. (2025). Engaging the Scientific Diaspora: The Case of Mexico. Barranquilla: Technical report, Universidad Simón Bolívar and International Development Research Centre (IDRC). doi: 10.17081/r.report.2025.11.17124

[B20] Instituto Centroamericano de Estudios Fiscales (ICEFI) and Oxfam Guatemala (2015). La Corrupción: sus Caminos, Su Impacto En La Sociedad y Una Agenda Para Su Eliminación. Guatemala: Technical report, ICEFI and Oxfam. Identifies budget lines vulnerable to corruption representing 29% of the 2015 total budget (Q20,814 million), and estimates that a 20% loss rate on those lines implies annual losses of approximately Q4.16 billion (6% of total budget). Available online at: https://www-cdn.oxfam.org/s3fs-public/file_attachments/ints._la_corrupcion._sus_caminos_su_impacto_en_la_sociedad_y_una_agend.pdf (Accessed March 23, 2026).

[B21] International Monetary Fund (2024). IMF Staff Country Reports no.: 2024(267). Guatemala: improving Social Spending to Boost Inclusive Economic Growth. doi: 10.5089/9798400287459.002

[B22] Ministerio Público de Guatemala (2016). Caso Lago de Amatitlán: MP, CICIG y el MINGOB Coordinan Captura de 14 Personas Vinculadas a Fraude Millonario. Guatemala City: Ministerio Público. Available online at: https://www.mp.gob.gt/noticia/caso-lago-de-amatitlan-mp-coordina-captura-de-14-personas-vinculadas-a-fraude-millonario/, Published 23 February 2016 (Accessed March 23, 2026).

[B23] Moshtari M. Ghorbani M. (2025), Challenges strategies for academic diaspora engagement in the internationalisation of higher education institutions: empirical evidence from a low- medium-income country. Higher Educ. Q. 79:e70004. 10.1111/hequ.70004

[B24] Newland K. Patrick E. (2004). Beyond Remittances: the Role of the Diaspora in Poverty Reduction in their Countries of Origin. Washington, DC: Migration Policy Institute. Migration Policy Institute. Available online at: https://www.migrationpolicy.org/sites/default/files/publications/Beyond_Remittances_0704.pdf (Accessed March 23, 2026).

[B25] No-Ficción (2020). Entrevista Edwin Asturias. Coronavirus. Guatemala City: No-Ficción. Available online at: https://no-ficcion.com/entrevista-edwin-asturias-coronavirus-guatemala/ (Accessed April 13, 2026)

[B26] No-Ficción (2025). Guatemala Sin Cientí*ficos?* Available online at: https://no-ficcion.com/guatemala-sin-cientificos/ (Accessed March 23, 2026).

[B27] OIM-MINEX International Organization for Migration (IOM) and Ministry of Foreign Affairs of Guatemala (MINEX) (2022). Ministry of Foreign Affairs (MINEX) and Organization of International Migration OIM. Guatemala: International Migration Review Forum. Technical report, MINEXand OIM. Available online at: https://www.un.org/sites/un2.un.org/files/imrf-guatemala_spanish.pdf (Accessed March 23, 2026).

[B28] Piñeros-Ayala R. (2025). The Colombian Scientific Diaspora: creating Bridges of Knowledge. Barranquilla: Technical report, Universidad Simón Bolívar and International Development Research Centre (IDRC). doi: 10.17081/r.report.2025.11.17130

[B29] Pombo K. (2025). Engagement of the Argentine Scientific Diaspora: analysis of the RAICES Program Network of Argentine Researchers and Scientists Abroad. Barranquilla: Technical report, Universidad Simón Bolívar and International Development Research Centre (IDRC). doi: 10.17081/r.report.2025.11.17126

[B30] Secretariat of Planning and Programming of the Presidency (SEGEPLAN) (2026). Evaluation Report of the General Government Policy 2024–2028: Year 2025. Guatemala City: SEGEPLAN. Available online at: https://portal.segeplan.gob.gt/segeplan/wp-content/uploads/2026/02/Informe-de-Evaluacion-de-la-PGG-2024-2028-Ano-2025-oficio-entrega.pdf (Accessed March 23, 2026).

[B31] Shin G.-W. Caywood K. (2025). Countering brain drain through circulation and linkage: illustrations and lessons from China and India. Int. Migr. Rev. 59, 1–35. doi: 10.1177/01979183251371676

[B32] Shin G.-W. Gordon H. M. (2024). Toward a portfolio theory of talent development: Insights from financial theory, illustrations from the Asia-Pacific. World Dev. 184:106755. doi: 10.1016/j.worlddev.2024.106755

[B33] Tobar P. and Alfredo, L.. (2011). La educación superior en Guatemala en la primera década del siglo XXI. Innovac. Educ. 11, 69–80.

[B34] UISUNESCO Institute for Statistics (2024). Guatemala: education and Literacy Statistics. Data Series: educational Attainment, Enrolment, and Government Expenditure on Education as % of GDP. Montreal, QC: UNESCO Institute for Statistics. Available online at: https://uis.unesco.org/country/GT. (Accessed March 23, 2026).

[B35] United Nations General Assembly (2018). Global Compact for Safe, Orderly and Regular Migration. Resolution GA/RES/73/195. New York, NY: United Nations. Available online at: https://docs.un.org/en/a/res/73/195 (Accessed December 19, 2018).

[B36] US Department of the Treasury (2023). Press Release: Treasury Sanctions Former Guatemalan Government Official for Engaging in Public Corruption. Washington, DC: US Department of the Treasury. Available online at: https://home.treasury.gov/news/press-releases/jy1941 (Accessed April 12, 2026).

[B37] World Bank (2025). Research and Development Expenditure (% of GDP), Guatemala. Available online at https://data.worldbank.org/indicator/GB.XPD.RSDV.GD.ZS?locations=GT. UNESCO Institute for Statistics (UIS) data accessed via World BankWorld Development Indicators (Accessed March 23, 2026).

